# Case report: diagnostic challenges of primary central nervous system anaplastic large cell lymphoma, ALK-positive

**DOI:** 10.1007/s00381-025-06904-3

**Published:** 2025-07-23

**Authors:** Abigail E. Weaver, Timothy J. Williamson, Krishnan R. Iyengar, Robert A. Campbell, Michael J. Stuart

**Affiliations:** 1 Department of Neurosurgery, Queensland Children’s Hospital, South Brisbane, QLD 4101 Australia; 2 Pathology Queensland, Royal Brisbane and Women’s Hospital, Herston, QLD Australia; 3 College of Medicine and Dentistry, James Cook University, Townsville, QLD 4814 Australia

**Keywords:** Hydrocephalus, Ventriculoperitoneal, Ventricular, Cerebrospinal fluid, Pediatric

## Abstract

**Introduction:**

Primary central nervous system lymphoma in children is rare, and the highly variable radiological presentation presents a significant diagnostic challenge. The reported subtype: anaplastic large cell lymphoma, kinase positive (ALCL, ALK+) is an exceedingly rare entity, with the largest case series comprising only 34 cases. Involvement of the choroid plexus is particularly uncommon, with only one previously reported case arising in the lateral ventricle. This case report highlights the diagnostic and management challenges associated with this unusual presentation and lesion location.

**Case report:**

A 16-year-old male presented with a 3-week history of fever and vomiting followed by progressive neurological deterioration, including confusion and collapse. Brain computed tomography (CT) revealed significant vasogenic edema and a hyperdense lesion in the right lateral ventricle. Magnetic resonance imaging (MRI) demonstrated a lobulated contrast-enhancing choroid plexus lesion with entrapment of the temporal horn and midline shift. Biopsy confirmed the diagnosis of PCNS ALK + ALCL. Subsequent chemotherapy resulted in remission of the lesion by the time of last follow-up, and aggressive surgical resection was not required. The rarity of this lesion posed diagnostic difficulties clinically and radiologically.

**Conclusion:**

This case illustrates the challenges of diagnosing childhood PCNSL, particularly when the lesion arises in an atypical location such as the choroid plexus. The excellent response to adjuvant therapy highlights the importance of considering this diagnosis in young patients with atypical brain lesions in order to avoid unnecessarily aggressive and potentially morbid surgical approaches.

## Introduction

Primary central nervous system lymphomas (PCNSL) represent fewer than 1.5% of all intracranial malignancies, with T-cell lymphomas comprising only 8.5% of these cases [1]. The ALK-positive (ALK +) variant of central nervous system ALCL is exceedingly rare, with the largest case series, published in 2022, reporting just 34 cases [2]. Nine reported cases have occurred in children [3]. These cases typically respond well to chemotherapy and generally do not require aggressive surgical resection, especially when located in deep or challenging-to-access locations [4]. For this reason, considering the diagnosis of lymphoma is important when developing a surgical strategy to avoid unnecessary surgical morbidity. While PCNSL is commonly considered in the differential diagnosis of adult lesions, this diagnosis is rarely encountered by pediatric neurosurgeons. Lesions which do not present with the typical “smudge-like” periventricular parenchymal enhancement may not necessarily trigger consideration of this diagnosis.

This case report highlights the diagnostic challenges of PCNS ALCL, attributed to its myriad clinical/radiological presentations and limited pediatric experience.

## Case report

A 16-year-old male with autism spectrum disorder and mild intellectual impairment presented to the emergency department with headache and confusion after a bout of fecal incontinence.

**Fig. 1:**
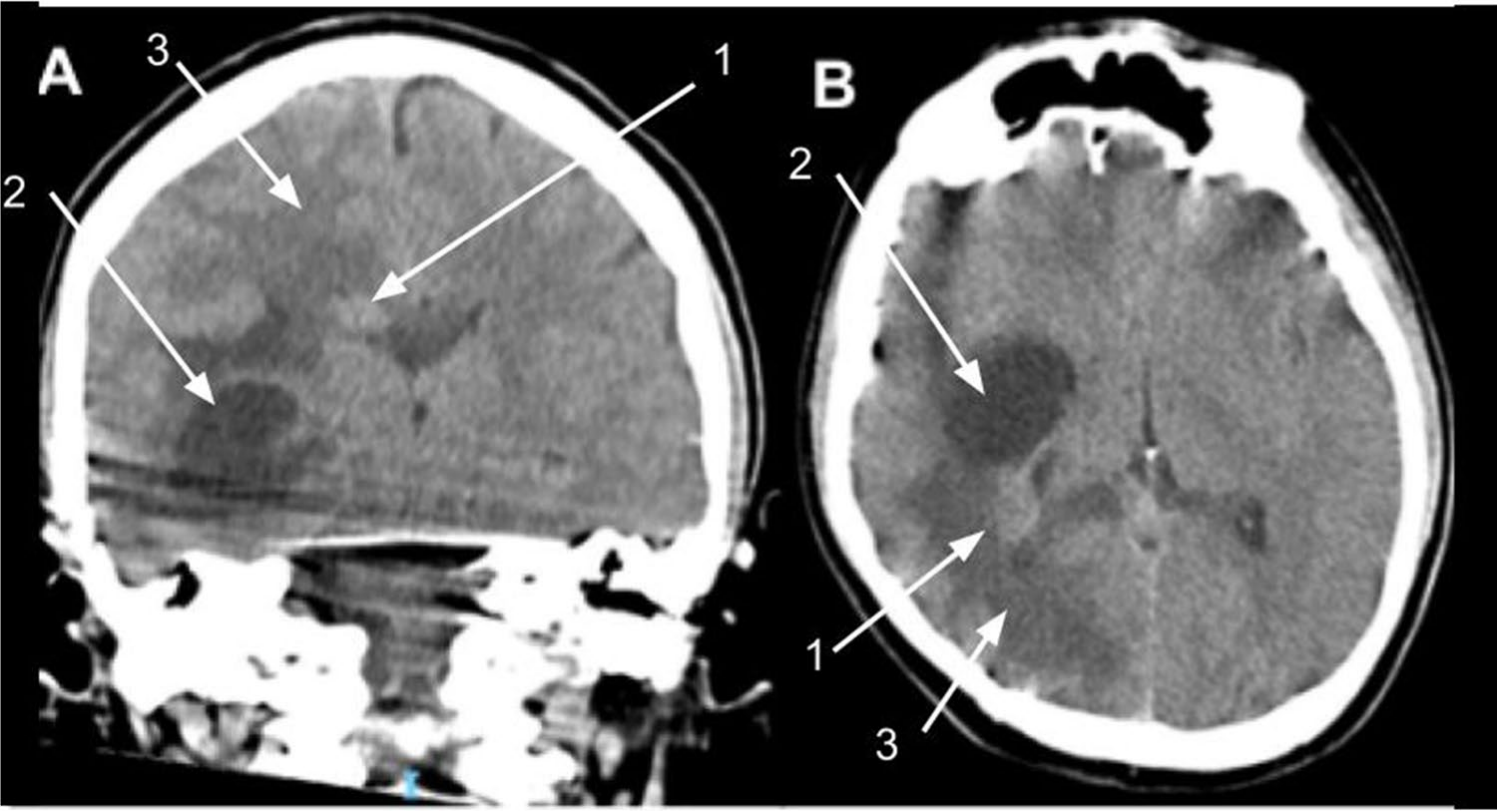
Computed tomography (CT) non-contrast brain images. **A** Coronal—mildly hyperdense infiltrating mass (1) in right lateral ventricle with apparent cystic temporal lobe component (2) and perilesional/ventricular edema (3). **B** axial—same lesion

He presented with a 3-week history of gastrointestinal symptoms and fever. The assessment in the emergency department raised concerns relating to persistent confusion, gait disturbance, and cephalgia. On examination, he was febrile with a Glasgow Coma Score of 14 and no focal neurological deficits. Laboratory findings revealed leukocytosis, neutrophilia, and an elevated C-reactive protein (CRP) level. Electrolytes were normal. The clinical presentation raised concern for an intracranial abscess with intraventricular rupture. Empiric broad-spectrum antibiotics (vancomycin and meropenem) and anti-seizure therapy (levetiracetam) were initiated. The patient was subsequently transferred to a specialized pediatric neurosurgical center for further evaluation and management (Fig. 1).

**Fig. 2:**
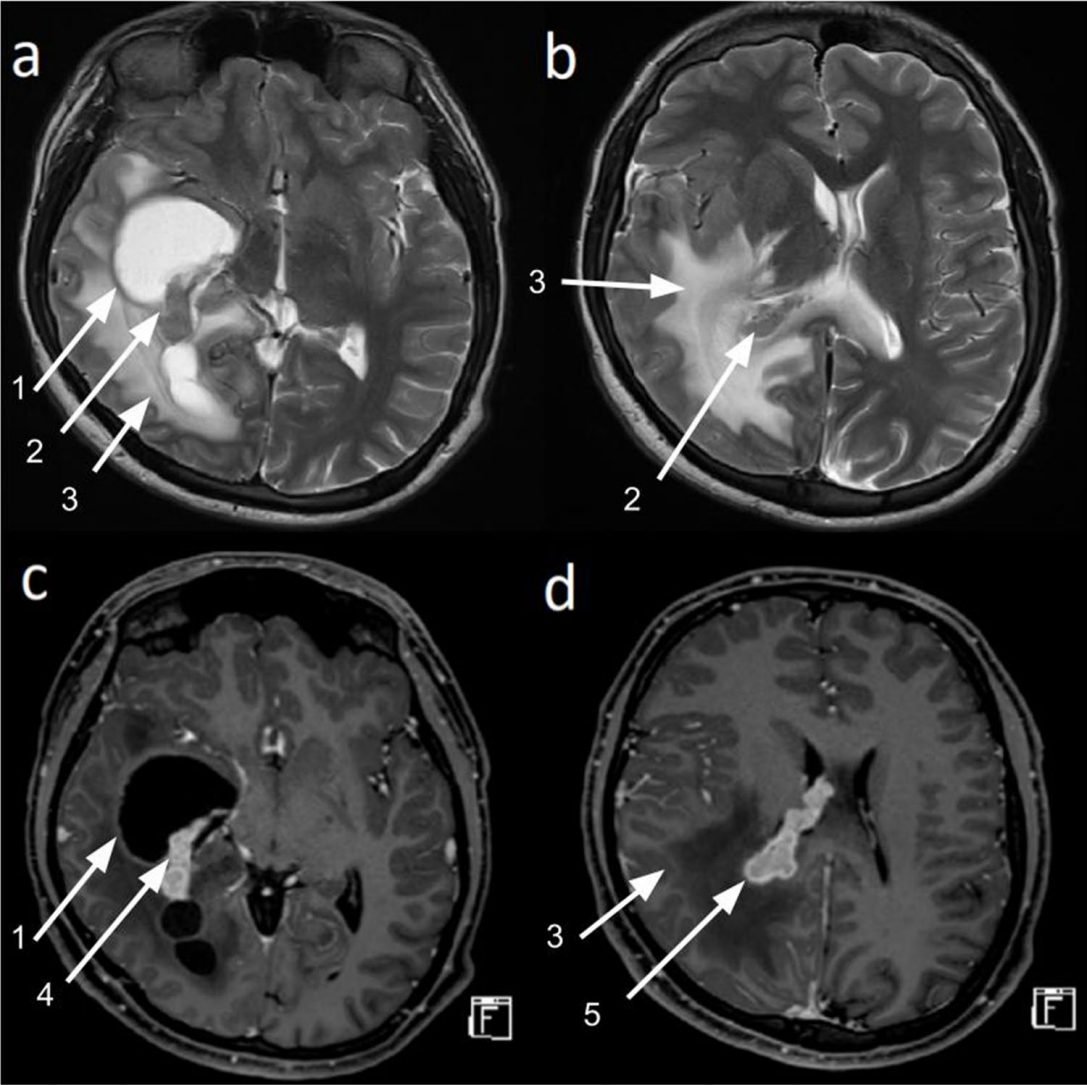
Preoperative magnetic resonance imaging (MRI). **a** Axial T2: Right-sided trapped temporal horn (1) with intraventricular lesion (2). **b** Axial T2: Periventricular edema (3)—right temporal and occipital lobe. **c** Axial T1 + contrast: Contrast-enhancing lesion within the trapped temporal horn (4). **d** Axial T1 + contrast: Contrast-enhancing lesion arising from the right choroid plexus (5)

MRI on arrival showed an irregular brightly enhancing lesion arising from the choroid plexus of the right lateral ventricle (Fig. 2). The lesion involved the posterior aspect of the corpus callosum. There was leptomeningeal enhancement across both hemispheres, with parenchymal involvement in the ipsilateral temporal lobe and bilateral occipital lobes. Extensive vasogenic edema was noted in the right parietal, occipital, and temporal lobes. The right temporal horn was trapped with 10 mm of midline shift and evidence of transtentorial herniation. The lesion was isointense to gray matter on both T1 and T2 sequences, with homogeneous post-contrast enhancement and patchy low signal on susceptibility-weighted imaging (SWI) consistent with hemorrhage. No diffusion restriction was noted (Fig. 2). At this stage, the infective presentation was considered to be a “red herring” and the differential diagnosis readjusted to neoplastic lesions, including primary lesions of the choroid plexus, germ cell tumors, and secondary malignancies.

The patient underwent urgent craniotomy for decompression of the temporal horn and biopsy of the intraventricular mass. Intraoperatively, the lesion was noted to be extremely vascular, in keeping with a lesion of the choroid plexus. An external ventricular drain (EVD) was placed in the temporal horn at the completion of the procedure. Postoperatively, high-dose dexamethasone improved his confusion, and the EVD was successfully weaned without need for further CSF diversion. His postoperative recovery was uneventful.

**Fig. 3:**
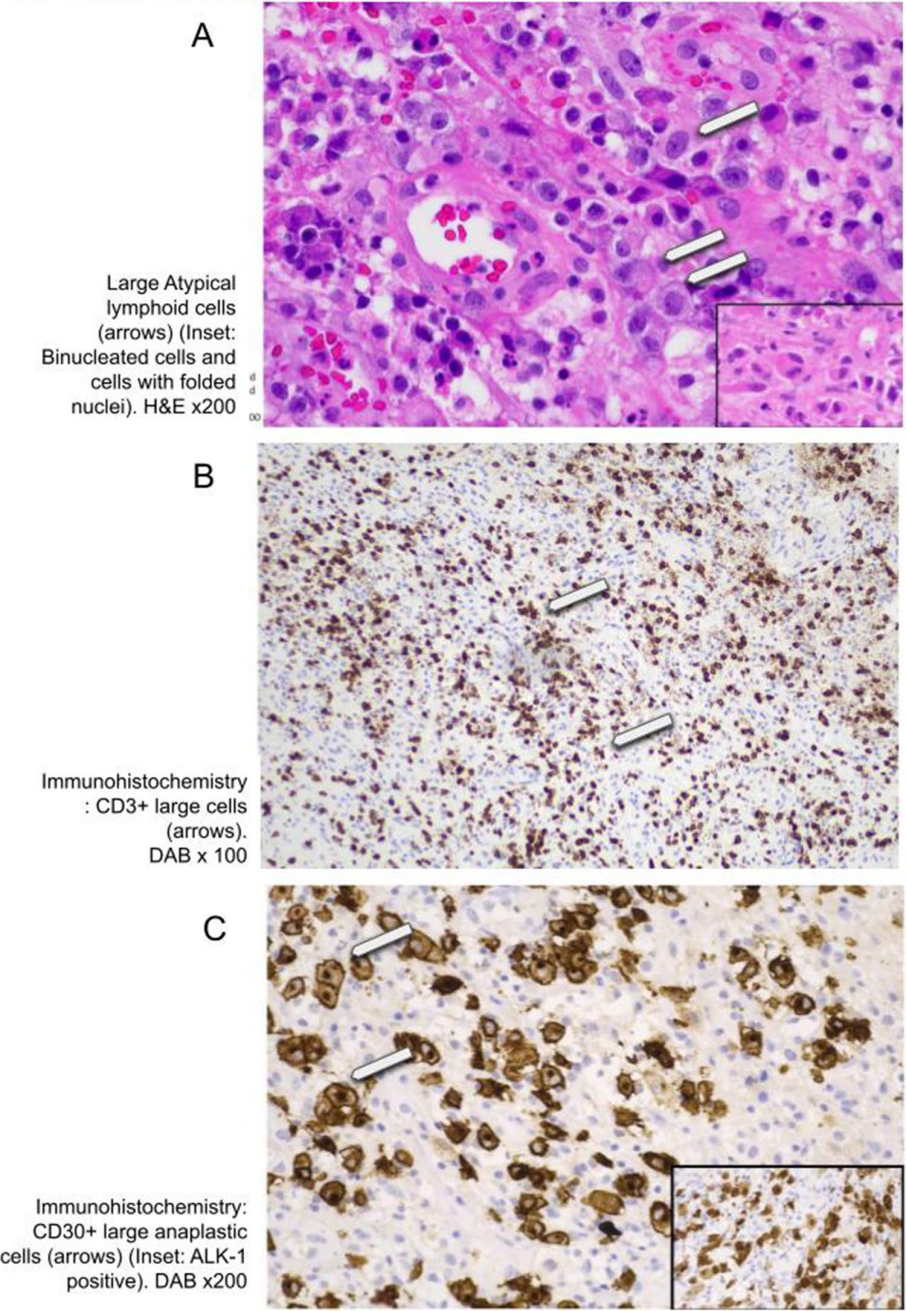
Brain biopsy histopathology

Histopathological analysis of the tissue biopsy exhibited characteristics consistent with ALCL ALK +. Histopathology revealed a polymorphous tumor composed of moderately pleomorphic large, atypical cells with vesicular nuclei and prominent nucleoli interspersed with neutrophils, eosinophils, histiocytes, and lymphocytes (Fig. 3A). The large cells demonstrated scattered mitotic figures including atypical mitoses. Occasional binucleated and multinucleated cells were seen (Fig. 3A). A rare cell with a bean-shaped nucleus was present, but classical Reed-Sternberg cells were not present. The atypical cells demonstrated CD45 and CD3 positivity as well as CD30 immunohistochemistry (Fig. 3B, C)). ALK immunohistochemistry was also positive (Fig. 3C). There was a Ki67 proliferation index of 50% focally.

Cerebrospinal fluid (CSF) analysis showed no malignant cells or tumor markers. Peripheral blood film and flow cytometry ruled out leukemia or systemic lymphoma. PET scan, skin biopsy, and bone marrow aspiration confirmed no extracranial involvement.

**Fig. 4:**
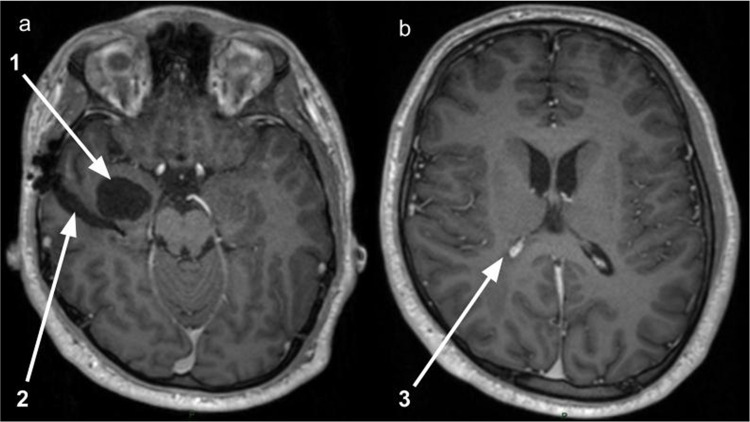
Magnetic resonance imaging (MRI)—post four doses of methotrexate. **a** Axial T1 + contrast: reduction in size of trapped temporal horn (1), operative tract posterior (2), resolution of leptomeningeal disease. **b** Axial T1 + contrast: reduction in contrast-enhancing lesion arising from the right choroid plexus (3)

Three months postoperatively, the patient completed four cycles of high-dose methotrexate. MRI showed significant reduction in lesion size and enhancement, with resolution of leptomeningeal involvement (Fig. 4). He remains clinically well and is initiating alectinib.

## Discussion

A Medline/PubMed search using MeSH terms “primary central nervous system neoplasm,” AND “T-cell lymphoma,” OR “anaplastic large cell lymphoma,” OR “CD30 + lymphoma” (past 10 years, English) identified two recent reviews: one on 34 cases of PCNS ALCL ALK + [2] and another from 2023 on radiological and histopathological findings in 17 cases [1].

ALK-positive ALCL is a rare primary CNS lymphoma with limited reported cases. A 2022 study of 34 patients found a median age of 18.5 years, mean of 20.6, and a male-to-female ratio of 4.7:1. Most lesions were solitary (73.5%) and supratentorial (94.1%) [2]. Our patient, near the median age, had a supratentorial lesion causing headache; however, its origin in the lateral ventricle with metastatic spread was rare, with only one other reported case of lateral ventricular involvement [5].

PCNS ALCL ALK + lacks distinctive radiological features, often delaying diagnosis. Leptomeningeal involvement can mimic meningitis or inflammation as seen in this case. A 2023 study of 17 cases found MRI commonly showed contrast enhancement (10/17), perilesional edema (9/17), and leptomeningeal enhancement (6/17). While no definitive radiological pattern has been established, lesions predominantly affect the cerebral hemispheres (82.3%) [1].

The authors recommend that in cases of unusual clinical and radiological presentations such as this, the initial strategy should be minimally invasive in order to obtain a histological diagnosis rather than pursuing a more aggressive resection strategy upfront. This allows for the appropriate treatment of such pathologies with chemotherapy and minimizes surgical morbidity. Specifically, in this lymphoma subtype, the ALK mutation is associated with a favorable prognosis and potential for targeted therapy with ALK inhibitors such as alectinib [6].

## Conclusion

PCNS ALCL ALK + is a rare diagnosis with nonspecific radiological features that can mimic other conditions. Further reports are needed to refine radiological recognition.

## Data Availability

No datasets were generated or analysed during the current study.
